# Increased co-expression of TIM-3 with TIGIT or 2B4 on CD8+ T cells is associated with poor prognosis in locally advanced nasopharyngeal carcinoma

**DOI:** 10.17305/bb.2022.8562

**Published:** 2023-08-01

**Authors:** Xiaolin Xie, Yaning Feng, Peiwen Fan, Danning Dong, Xuan Yao, Yanchun Peng, Ruozheng Wang

**Affiliations:** 1 The Third Affiliated Teaching Hospital of Xinjiang Medical University, Affiliated Cancer Hospital, Urumqi, China; 2 Key Laboratory of Cancer Immunoterapy and Radiotherapy, Chinese Academy of Medical Sciences, Affiliated Tumor Hospital of Xinjiang Medical University, Urumqi, China; 3 Xinjiang Key Laboratory of Oncology, Urumqi, Xinjiang, China; 4 Key Laboratory of Oncology of Xinjiang Uyghur Autonomous Region, Urumqi, China; 5 CAMS Oxford Institute (COI), University of Oxford, Oxford, United Kingdom

**Keywords:** Locally advanced nasopharyngeal carcinoma (NPC), inhibitory receptor, co-expression, prognosis

## Abstract

The use of immune checkpoint inhibitors in malignant tumors improves patient outcomes. Because single-agent immune checkpoint blockade has a low objective response rate, it is meaningful to explore the combined blockade of immune checkpoint receptors. We aimed to investigate the co-expression of TIM-3 with TIGIT or 2B4 on peripheral blood CD8+ T cells from patients with locally advanced nasopharyngeal carcinoma. The correlation between co-expression level and clinical characteristics and prognosis was studied to provide a basis for immunotherapy for nasopharyngeal carcinoma. Flow cytometry was used to detect TIM-3/TIGIT and TIM-3/2B4 co-expression on CD8+ T cells. The differences in co-expression between patients and healthy controls were analyzed. The correlation between the co-expression of TIM-3/TIGIT or TIM-3/2B4 and the patient clinical characteristics and prognosis was examined. Also, the correlation between the TIM-3/TIGIT or 2B4 co-expression and other common inhibitory receptors was analyzed. We further validated our results using mRNA data from the Gene Expression Omnibus (GEO) database. TIM-3/TIGIT and TIM-3/2B4 co-expression was upregulated on peripheral blood CD8+ T cells from patients with nasopharyngeal carcinoma. They were both correlated with poor prognosis. There was a correlation between TIM-3/TIGIT co-expression and patient age and pathological stage, whereas TIM-3/2B4 co-expression correlated with age and sex. CD8+ T cells with elevated mRNA levels of *TIM3/TIGIT* and *TIM3/2B4* also showed increased expression of multiple inhibitory receptors, indicating T cell exhaustion in locally advanced nasopharyngeal carcinoma. TIM-3/TIGIT or TIM-3/2B4 can be used as potential targets for combination immunotherapy in locally advanced nasopharyngeal carcinoma.

## Introduction

Nasopharyngeal carcinoma (NPC) is one of the most common malignant tumors of the head and neck, originating from the mucosal epithelium of the nasopharynx. NPC is closely related to Epstein–Barr virus infection. The latest data from the National Cancer Centre reveal that the number of new cases of NPC in China is 52,000, while the number of deaths is 27,000 [1]. Concurrent chemotherapy and intensity-modulated radiotherapy are currently the main treatment for locally advanced NPC. However, the local recurrence still occurs in 5%–15% of the patients and distant metastases are seen in 15%–30% of patients after treatment [2]. Therefore, there is a need for exploratory studies to provide evidence for a wider choice of treatment options.

In recent years, immune checkpoint inhibitors (ICIs) have emerged as the mainstay of immunotherapy. Expression of immune checkpoint receptors leads to T cell exhaustion or function suppression, thus promoting tumorigenesis and progression, while immune checkpoint blocking can improve patient prognosis by targeting immune checkpoint receptors [2, 3]. Despite the fact that programmed cell death protein 1 (PD-1) inhibitors are effective in recurrent/metastatic NPC, the objective response rate is still poor at 22.5% [4, 5], and resistance to a single ICI therapy may develop over time. As a result, there is an urgent need to explore combination therapies for immunotherapy to enhance the efficacy and benefit more patients [6].

A number of new inhibitory receptors, such as T cell immunoglobulin and ITIM domain (TIGIT), T cell immunoglobulin domain and mucin domain-3 (TIM-3), and the cluster of differentiation 244 (CD244 or 2B4), have emerged as potential candidates for the treatment of advanced malignancies [7]. Several studies reported that TIM-3, TIGIT, and 2B4 are strongly associated with T cell exhaustion. TIM-3 is identified as a molecule expressed on the surface of T cells [8]. TIM-3 is an important immune checkpoint receptor mediating T cell exhaustion, thus suppressing anti-tumor immunity [9]. In vitro assays showed that TIM-3 can affect the migration and invasion of NPC cells through SMAD7/SMAD2/SNAIL1 signaling pathway [10]. TIGIT is expressed on the surface of all types of T cells and natural killer (NK) cells [11] and inhibits T cell activation by mediating inhibitory signals [12, 13]. TIGIT was shown to be a good therapeutic target in a variety of tumors [14]. 2B4 is mainly expressed on the surface of NK cells, T cells, etc. When it binds to its ligand CD48, 2B4 can generate inhibitory and activating signals, playing an important regulatory role in tumors [15].

Several ongoing clinical trials have evaluated the effect of integrating TIM-3 inhibitor into existing treatment of cancer, including the combination of the anti-TIM-3 antibody sabatolimab with the anti-PD-1 antibody spartalizumab in advanced solid tumors, which showed good anti-tumor efficacy and acceptable tolerability [16]. In a study on esophageal cancer, co-expression of TIM-3 and programmed death ligand protein 1 (PD-L1) on T cells was found to be an independent prognostic factor [17]. Targeting TIM-3 together with other inhibitory receptors seems to be a promising therapeutic strategy with potential clinical applications. However, to our knowledge, there are currently few studies on co-expression patterns of inhibitory receptors in NPC. Therefore, in this study, we investigated the co-expression of TIM-3 with TIGIT or 2B4 on peripheral CD8+ T cells in patients with NPC. We further explored the relationship between the co-expression levels of TIM-3 with TIGIT or 2B4 and clinical features as well as prognosis in NPC, hoping to provide evidence for the potential use of dual inhibition of TIM-3 and TIGIT or 2B4 as a combination treatment strategies for NPC.

## Materials and methods

### Source of specimens

Treatment-naive patients with locally advanced NPC admitted to the Head and Neck Radiotherapy Department of the Affiliated Cancer Hospital of Xinjiang Medical University between August 2016 and April 2021 were selected for the study. The patients were treated with radiotherapy. Relevant clinical information was collected. Forty-four healthy subjects were selected as healthy controls.

Inclusion criteria for patients were: (1) histopathologically confirmed NPC and not been treated for NPC when recruited to our study; (2) Karnofsky score ≥ 80; (3) age ≥ 18 years; and (4) complete serological and clinical data. Exclusion criteria were: (1) patients with stage I and II NPC or distant metastases; (2) having other malignancies or a history of any malignancies; and (3) diagnosis of other diseases, such as autoimmune diseases, chronic infections, infectious diseases, etc. Eighty-three patients were finally selected. The cohort included 60 males and 23 females, aged 18–81 years (median 52 years). There were 35 smokers and 48 non-smokers. Twenty-three patients had non-keratinizing differentiated carcinoma, 45 had non-keratinizing undifferentiated carcinoma, and 15 had other types. All patients were clinically staged according to the Union for International Cancer Control and American Joint Committee on Cancer (UICC/AJCC) 8th edition using the TNM staging of NPC.

### Treatment

All patients received standard treatment according to the National Comprehensive Cancer Network (NCCN) guidelines. Prior to radiotherapy, 92.8% of patients received 2–3 cycles of TP (docetaxel and cisplatin) or GP (gemcitabine and cisplatin) regimens of induction chemotherapy. TP consisted of docetaxel (75 mg/m^2^, day 1) + cisplatin (75 mg/m^2^, days 1–3) and GP consisted of gemcitabine (1 g/m^2^, day 1 and day 8) + cisplatin (75 mg/m^2^, days 1–3). All patients received concurrent radiotherapy with cisplatin regimens. The regimen was 30–40 mg/m^2^ for 6–7 cycles. The radiotherapy modality was conformal intensity-modulated radiotherapy with a total prescribed dose of 66–69.96 Gy, with primary gross tumor volume (PGTVnx) 69.96 Gy, gross tumor volume of the lymph nodes (GTVnd) 69.96 Gy, planning target volume (PTV) 160.06 Gy, and PTV 250.96 Gy.

### Peripheral blood mononuclear cells isolation

Peripheral blood mononuclear cells (PBMCs) were isolated by density gradient centrifugation as previously described [18] using 10 mL of peripheral blood collected in EDTA tubes and counted in trypan blue.

### Flow cytometry

0.6 × 10^6^ PBMCs were washed with phosphate-buffered saline (PBS) (Sigma) and first stained with LIVE/DEAD™ Fixable Aqua Dead Cell Stain (Life Technologies) for 20 min, followed by surface staining with mouse anti-human CD3-AF700 antibody, mouse anti-human CD8-APC-H7 antibody, mouse anti-human CD4-FITC antibody, mouse anti-human TIGIT-PE-A antibody, mouse anti-human TIM-3-BB515 antibody and 2B4-APC antibody, CTLA4, PD-1, and KLRG1 (BD) for 20 min. Finally, 300 µL FACS fixing buffer (BD) was added to fix the cells for acquisition by flow cytometry. All data were acquired on BD LSRFortessa™ Cell Analyzer (BD).

### Data sources

The gene expression profiles used in this study were downloaded from the US Biotechnology Information Center GEO database (https://www.ncbi.nlm.nih.gov/geo/). We selected three microarray datasets (GSE12452, GSE64634, and GSE61218). The GSE12452 and GSE64634 datasets were both generated on the GPL570 sequencing platform, and the GSE61218 dataset was generated on the GPL19061 platform. The batch effect was corrected using the ComBat function from the sva package [19].

**Figure 1.**
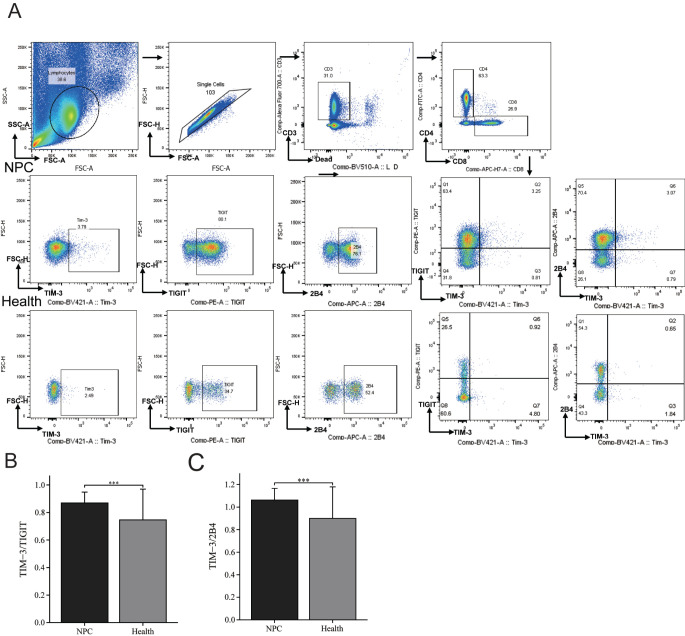
**Gating strategy to identify TIM-3/TIGIT or TIM-3/2B4 double positive CD8+ T cells in PBMC using flow cytometry.** (A) PBMCs were stained and examined on flow cytometer. Representative flow cytometry plots from NPC patients and healthy subjects are shown for the identification of TIM-3+ CD8+ T cells, TIGIT+ CD8+ T cell, 2B4+ CD8+ T cells, TIM-3+ TIGIT+ CD8+T cells, and TIM-3+ 2B4+ CD8+ T cells. Comparison of the expression levels of (B) TIM-3/TIGIT and (C) TIM-3/2B4 on CD8+ T cells from patients with nasopharyngeal carcinoma and healthy controls. (ns, *P* ≥ 0.05, *P < 0.05, **P < 0.01, ***P < 0.001). PBMCs: Peripheral blood mononuclear cells; NPC: Nasopharyngeal carcinoma; Health: Healthy controls.

### Follow-up

Overall survival (OS) was defined as the time from the date of diagnosis to the last follow-up visit or death from any cause. Progression-free survival (PFS) was defined as the time from the date of diagnosis to the date of the first tumor recurrence, distant metastasis, or death from any cause. Distant metastasis-free survival (DDFS) was defined as the time from the date of diagnosis to the date of first distant metastasis or death from any cause.

### Ethical statement

The study was approved by the Ethics Committee of the Affiliated Cancer Hospital of Xinjiang Medical University (the number of approval: K-2022038). All study subjects signed an informed consent form.

### Statistical analysis

The data were analyzed using the SPSS 26.0 software package. Comparisons of count data were made using the chi-square test or Fisher’s exact probability test. The *t*-test was used for the comparison of quantitative data. The rank sum test was used for comparison of data that did not meet the normality. OS, PFS, and DDFS were estimated using the Kaplan–Meier method, with one-way analysis Log-rank test, and Cox proportional risk regression model for multi-factor analysis. R (version 3.6.3) was used for statistical analysis and visualization, ggplot2 [version 3.3.3] (for visualization). Receiver operating characteristic (ROC) curves for TIM-3/TIGIT or TIM-3/2B4 co-expression were created and the best cutoff values were determined by the Jorden index. Differences with *P* values less than 0.05 were considered statistically significant.

**Table 1:** Relationship between TIM-3/TIGIT or TIM-3/2B4 co-expression on peripheral CD8+ T cells and clinical features in 83 patients with nasopharyngeal carcinoma

**Characteristics**	**TIM-3/TIGIT high co-expression**	**TIM-3/TIGIT low co-expression**	***P***	**TIM-3/2B4 high co-expression**	**TIM-3/2B4 low co-expression**	***P***
Sex, *N* (%)						
Male	29 (34.9)	31 (37.4)	0.084	37 (44.6)	23 (27.7)	0.014
Female	6 (7.2)	17 (20.5)		7 (8.4)	16 (19.3)	
Age (years), *N* (%)						
<52	12 (14.5)	29 (34.9)	0.026	15 (18.1)	26 (31.3)	0.004
≥52	23 (27.7)	19 (22.9)		29 (34.9)	13 (15.7)	
Smoking, *N* (%)						
Yes	19 (22.9)	16 (19.3)	0.073	23 (27.7)	12 (14.5)	0.074
No	16 (19.3)	32 (38.6)		21 (47.7)	27 (32.5)	
Pathology, *N* (%)						
NKDC	5 (6.0)	18 (21.7)	0.045	10 (12.0)	13 (15.7)	0.192
NKUC	21 (25.3)	24 (28.9)		23 (27.7)	22 (26.5)	
Other	9 (10.9)	6 (7.2)		11 (13.3)	4 (4.8)	
Clinical stage, *N* (%)						
III	23 (27.7)	32 (38.5)	1.000	29 (34.9)	26 (31.3)	1.000
IVA	12 (14.5)	16 (19.3)		15 (18.1)	13 (15.7)	
T stage, *N* (%)						
T1–T2	9 (10.9)	8 (9.6)	0.484	10 (12.1)	7 (8.4)	0.714
T3	18 (21.7)	31 (37.3)		24 (28.9)	25 (30.1)	
T4	8 (9.6)	9 (10.9)		10 (12.1)	7 (8.4)	
N stage, *N* (%)						
N0–N1	4 (4.8)	7 (8.4)	0.725	6 (13.6)	5 (12.8)	0.730
N2	27 (32.5)	33 (39.8)		33 (75.0)	27 (69.2)	
N3	4 (4.8)	8 (9.6)		5 (11.4)	7 (17.9)	

NKDC: Nonkeratinizing differentiated carcinoma; NKUC: Nonkeratinizing undifferentiated carcinoma.

## Results

### TIM-3/TIGIT and TIM-3/2B4 co-expression on CD8+ T cells in nasopharyngeal carcinoma patients was higher than in healthy controls

First, we compared the co-expression of TIM-3 with TIGIT or 2B4 on CD8+ T cells in peripheral blood of the 83 patients with NPC and 44 healthy subjects. The gating strategy is shown in Figure 1A. The frequency of TIM-3/TIGIT double positive CD8+ T cells was 0.7% (interquartile range [IQR] 0.365%–1.07%) in the NPC group, higher than in the healthy control (0.33% [IQR 0.238%–0.502%]). Comparative analysis showed that NPC patients had higher co-expression than healthy controls, with a significant median difference of 0.29% (IQR 0.13%–0.46%; *P* < 0.001) between the two groups (Figure 1B). On the other hand, the frequency of TIM-3/2B4 double positive CD8+ T cells was 0.83% (IQR 0.5%–1.31%) in the NPC group and 0.365% (IQR 0.228%–0.635%) in healthy controls. Comparative analysis showed that NPC patients had higher co-expression than healthy controls, with a significant median difference of 0.33% (IQR 0.18%–0.51%; *P* < 0.001) between the two groups (Figure 1C). It can be seen that peripheral CD8+ T cells in NPC patients showed increased co-expression level of both TIM-3/TIGIT and TIM-3/2B4.

### Co-expression of inhibitory receptors TIM-3/TIGIT and TIM-3/2B4 on CD8+ T cells in relation to clinical features in NPC patients

We then investigated if the co-expression of TIM-3 with TIGIT or 2B4 on peripheral CD8+ T cells from NPC patients was related to any of the clinical features. Based on the ROC cutoff values, we classified patients into groups with high or low co-expression levels of TIM-3/TIGIT or TIM-3/2B4. Of the 83 patients with NPC, 35 (42.2%) showed high level of TIM-3/TIGIT co-expression, whereas 48 (57.8%) showed low level of co-expression. Forty-four (53.1%) patients showed high TIM-3/2B4 co-expression and 39 (46.9%) showed low level of co-expression. Of all clinical characteristics we investigated (Table 1), patients with high level of TIM-3/TIGIT or TIM-3/2B4 co-expression were predominantly ≥ 52 years old (23/83, 27.7% for TIM-3/TIGIT; 29/83, 34.9% for TIM-3/2B4), both with statistically significant differences (*P* < 0.05). When compared with the pathological staging, the patients with higher level of TIM-3/TIGIT co-expression predominantly had non-keratinizing undifferentiated carcinoma (21/83, 25.3%) and patients with low level of TIM-3/TIGIT co-expression also predominantly had non-keratinizing undifferentiated carcinoma (24/83, 28.9%), with statistically significant differences (Table 1). We also found that male patients tended to have higher level of TIM-3/2B4 co-expression (37/83, 44.6%) (Table 1).

### Co-expression of inhibitory receptors TIM-3/TIGIT or TIM-3/2B4 in CD8+ T cells in nasopharyngeal carcinoma was associated with poor prognosis

**Figure 2.**
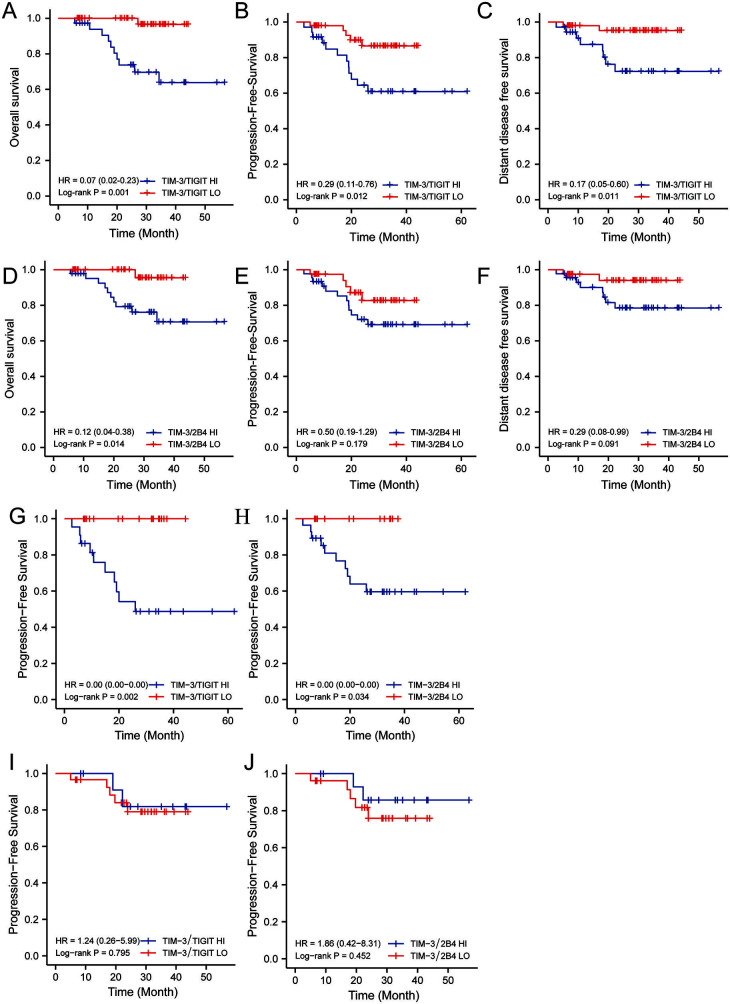
**Comparison of prognosis between patients with high or low level of TIM-3/TIGIT or TIM-3/2B4 co-expression**. Prognostic analysis of TIM-3/TIGIT co-expression levels for (A) OS, (B) PFS, and (C) DDFS. Prognostic analysis of TIM-3/2B4 co-expression levels for (D) OS, (E) PFS, and (F) DDFS. Prognostic analysis of (G) TIM-3/TIGIT and (H) TIM-3/2B4 co-expression levels and PFS in nasopharyngeal carcinoma patients aged ≥ 52 years. Prognostic analysis of (I) TIM-3/TIGIT and (J) TIM-3/2B4 co-expression levels and PFS in nasopharyngeal carcinoma patients aged < 52 years. HR: Hazard ratio; OS: Overall survival; PFS: Progression-free survival; DDFS: Distant metastasis-free survival; HI: High co-expression; LO: Low co-expression.

We looked into the relationship between the co-expression of the inhibitory receptors with the prognosis in NPC patients. Follow-up of 83 patients with NPC started at enrollment and continued until 1 November 2021, with a follow-up period ranging from 5.6 to 56.8 months. The median follow-up time for the 83 patients with NPC was 27.4 months, with an overall survival rate of 81.1% at 3 years for all patients. A 100% follow-up rate was maintained for all analyzed patients. Seventeen of the 83 patients with NPC had progression (20.5%), of whom 10 had distant metastases (12.5%) and 11 died (13.3%). We used Kaplan–Meier analysis to compare overall survival (OS), progression-free survival (PFS), and distant-disease-free survival (DDFS) between patients with different levels of inhibitory receptor co-expression. We noticed that the patients with higher level of TIM-3/TIGIT or TIM-3/2B4 co-expression showed significantly poorer prognosis (*P* ═ 0.014 for TIM-3/TIGIT; *P* ═ 0.008 for TIM-3/2B4) (Figure 2A and 2D). Patients with lower level of TIM-3/TIGIT co-expression displayed significantly better PFS (*P* ═ 0.012) (Figure 2B), which was not seen for TIM-3/2B4 (*P* ═ 0.179) (Figure 2E). Similarly, patients with lower level of TIM-3/TIGIT co-expression had better DDFS than those with higher co-expression (*P* ═ 0.017) (Figure 2C), whereas TIM-3/2B4 was not associated with DDFS (*P* ═ 0.091) (Figure 2F). In addition, the high level of co-expression of TIM-3 with either TIGIT or 2B4 was significantly associated with worse PFS in patients older than 52 years (Figure 2G and 2H), contrarily to younger patients (Figure 2I and 2J).

### TIM-3/TIGIT co-expression on CD8+ T cells was an independent prognostic factor for OS in nasopharyngeal carcinoma

We found that several factors were related to the OS in NPC patients. Univariate analysis showed that TIM-3/TIGIT co-expression (hazard ratio [HR] 6.445, 95% confidence interval [CI] 1.839–112.541; *P* ═ 0.011), TIM-3/2B4 co-expression (HR 8.601; 95% CI 1.101–67.205; *P* ═ 0.04), age (HR 3.816, 95% CI 1.032–14.115; *P* ═ 0.045), and smoking (HR 3.823, 95% CI 1.343–10.882; *P* ═ 0.012) were risk factors for OS in NPC patients (Table 2). Multiple factor analysis showed that TIM-3/TIGIT co-expression (HR 10.157, 95% CI 1.286–80.216; *P* ═ 0.028) was an independent prognostic factor related to the OS (Table 2). As shown in Table 3 (univariate analysis), both high level of TIM-3/TIGIT co-expression and smoking were statistically significantly associated with poor PFS. However, only smoking was an independent factor (HR 3.186, 95% CI 1.104–9.189; *P* ═ 0.032)

**Table 2:** Univariate and multiple factor analysis of TIM-3/TIGIT or TIM-3/2B4 co-expression on peripheral CD8+ T cells and overall survival in 83 patients with nasopharyngeal carcinoma

				**Multivariate**			
		**Univariate**		**TIM-3/TIGIT**		**TIM-3/2B4**	
**Variables**		***P***	**HR (95% CI)**	***P***	**HR (95% CI)**	***P***	**HR (95% CI)**
TIM-3/TIGIT	LO		1		1		
	HI	0.011	6.455 (1.839-112.541)	0.028	10.157 (1.286–80.216)		
TIM-3/2B4	LO		1				1
	HI	0.04	8.601 (1.101–67.205)			0.129	5.047 (0.625–40.725)
Age (years)	<52		1		1		1
	≥52	0.045	3.816 (1.032–14.115)	0.076	4.035 (0.863–18.874)	0.101	3.670 (0.776–17.361)
Sex	Male		1				
	Female	0.776	0.850 (0.277–2.608)				
Smoking	No		1		1		1
	Yes	0.012	3.823 (1.343–10.882)	0.093	3.133 (0.825–11.898)	0.082	3.275 (0.861–12.468)
Pathology	NKDC		1				
	NKUC	0.474	1.528 (0.478–4.878)				
	Other	0.560	1.562 (0.349–6.996)				
T stage	T1–2		1				
	T3	0.804	0.812 (0.157–4.191)				
	T4	0.396	2.086 (0.382–11.397)				
N stage	N0–1		1				
	N2	0.355	0.469 (0.094–2.333)				
	N3	0.665	1.485 (0.248–8.906)				
Clinical stage	III		1				
	IVA	0.131	2.399 (0.772–7.459)				

NKDC: Nonkeratinizing differentiated carcinoma; NKUC: Nonkeratinizing undifferentiated carcinoma; HR: Hazard ratio; CI: Confidence interval. HI: High co-expression; LO: Low co-expression

**Table 3:** Univariate and multiple factor analysis of TIM-3/TIGIT or TIM-3/2B4 co-expression on peripheral CD8+ T cells and progression-free survival in 83 patients with nasopharyngeal carcinoma

				**Multivariate**	
		**Univariate**		**TIM-3/TIGIT**	
**Variables**		***P***	**HR (95% CI)**	***P***	**HR (95% CI)**
TIM-3/TIGIT	LO		1		1
	HI	0.019	3.482 (1.226–9.885)	0.052	2.849 (0.990–8.196)
TIM-3/2B4	LO		1		
	HI	0.188	2.016 (0.710–5.725)		
Age (years)	<52		1		
	≥52	0.776	0.850 (0.277–2.608)		
Sex	Male		1		
	Female	0.373	0.501 (0.110–2.290)		
Smoking	No		1		1
	Yes	0.018	4.843 (1.306–17.956)	0.032	3.186 (1.104–9.189)
Pathology	NKDC		1		
	NKUC	0.318	2.206 (0.467–10.42)		
	Other	0.435	2.184 (0.307–15.542)		
T stage	T1–2		1		
	T3	0.789	1.193 (0.382–4.335)		
	T4	0.586	1.516 (0.339–6.780)		
N stage	N0–1		1		
	N2	0.95	0.953 (0.211–4.3)		
	N3	0.374	2.161 (0.395–11.827)		
Clinical stage	III		1		
	IVA	0.354	1.579 (0.600–4.153)		

NKDC: Nonkeratinizing differentiated carcinoma; NKUC: Nonkeratinizing undifferentiated carcinoma; HR: Hazard ratio; CI: Confidence interval; HI: High co-expression; LO: Low co-expression.

### CD8+ T cells with elevated level of TIM-3/TIGIT or TIM-3/2B4 co-expression showed exhausted phenotype of CD8+ T cells in NPC patients

**Figure 3.**
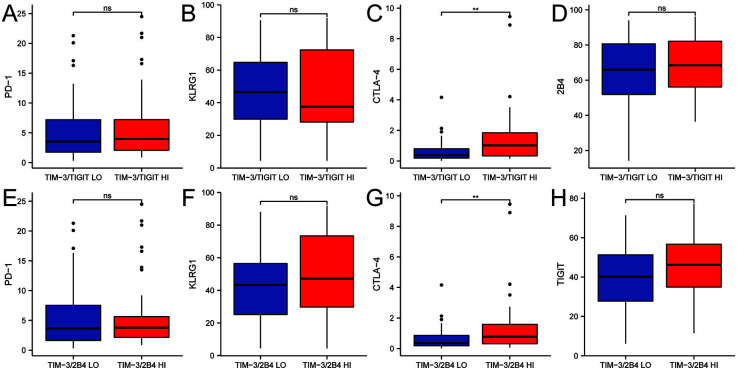
Comparison of expression levels of (A) PD-1, (B) KLRG1, (C) CTLA-4, and (D) 2B4 on peripheral blood CD8+ T cells of the 83 NPC patients with high or low level of TIM-3/TIGIT co-expression. Comparison of expression levels of (E) PD-1, (F) KLRG1, (G) CTLA-4, and (H) TIGIT on peripheral blood CD8+ T cells of the 83 NPC patients with high or low level of TIM-3/2B4 co-expression (ns, *P* ≥ 0.05, *P < 0.05, **P < 0.01, ***P < 0.001). PD-1: Programmed cell death protein 1; KLRG1: Killer cell lectin-like receptor subfamily G member 1; CTLA-4: Cytotoxic T-lymphocyte-associated protein 4; NPC: Nasopharyngeal carcinoma; HI: High co-expression; LO: Low co-expression.

To further explore if the co-expression of TIM-3 with TIGIT or 2B4 was related to the exhausted phenotype, we compared the levels of a few other inhibitory receptors on CD8+ T cells in NPC patients with high or low level of co-expression. The results showed that cytotoxic T-lymphocyte-associated protein 4 (CTLA-4) expression was significantly higher in patients with higher level of TIM-3/TIGIT co-expression than in the patients with lower co-expression level, with a median difference of 0.46 (IQR 0.143-0.88; *P* ═ 0.001) between the two groups (Figure 3C). PD-1 (median difference 0.275) (Figure 3A), killer cell lectin-like receptor subfamily G member 1 (KLRG1) (median difference 0.5) (Figure 3B), and 2B4 (mean difference 3.36) (Figure 3D) were not different between the two groups. Similarly, CTLA-4 expression was also found to be significantly higher in patients with high level of TIM-3/2B4 co-expression than in the patients with TIM-3/2B4 low co-expression, with a median difference of 0.3 (IQR 0.08-0.661; *P* ═ 0.006) between the two groups (Figure 3G). However, PD-1 (median difference 0.2) (Figure 3E), KLRG1 (median difference 0.2) (Figure 3F), and TIGIT (mean difference 6.072) (Figure 3H) were not different between the two groups.

**Figure 4.**
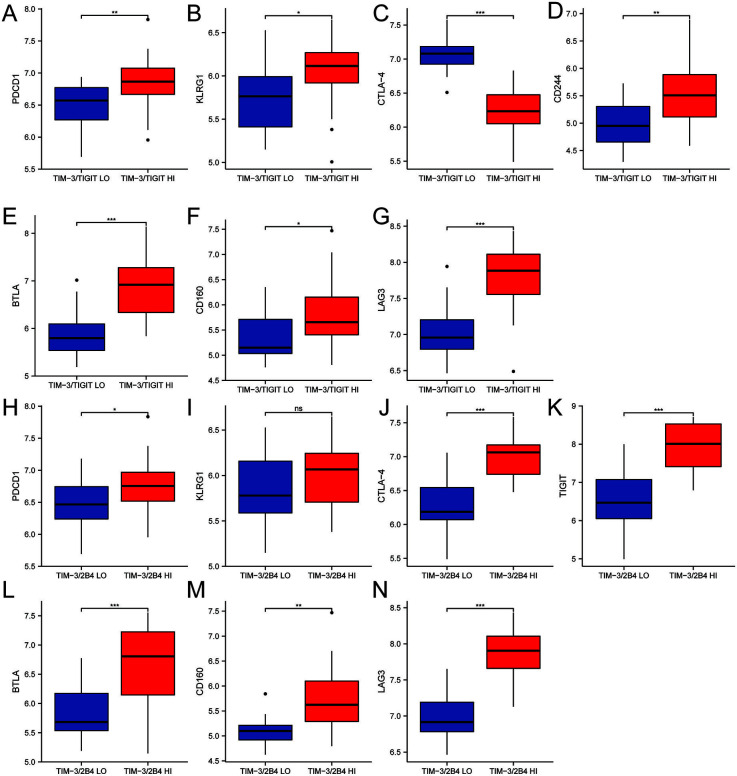
Comparison of mRNA levels of (A) *PDCD1*, (B) *KLRG1*, (C) *CTLA4*, (D) *CD244*, (E) *BTLA*, (F) *CD160*, and (G) *LAG3* of the 53 NPC patients in the GEO database with high or low level of *TIM3/TIGIT* co-expression. Comparison of mRNA levels of (H) *PDCD1*, (I) *KLRG1*, (J) *CTLA4*, (K) *CD244*, (L) *BTLA*, (M) *CD160*, and (N) *LAG3* the 53 NPC patients in the GEO database with high or low level of *TIM3/2B4* co-expression (ns, *P* ≥ 0.05; *P < 0.05; **P < 0.01; ***P < 0.001). PDCD-1: Programmed cell death protein 1; KLRG1: Killer cell lectin-like receptor subfamily G member 1; CTLA-4: Cytotoxic T-lymphocyte-associated protein 4; LAG-3: Lymphocyte activation gene 3; NPC: Nasopharyngeal carcinoma; HI: High co-expression; LO: Low co-expression.

To validate our findings in a different cohort, we extracted data of 53 patients with NPC from the GEO database and analyzed the correlation of *HAVCR2* (*TIM3*), *TIGIT*, *2B4*, *PDCD*, *KLRG1*, *CTLA4*, *CD244*, *BTLA*, *CD160*, and *LAG3* on the mRNA level. We classified 53 patients into the subgroups with high or low level of *HAVCR2/TIGIT* or *HAVCR2/2B4* co-expression according to the median. We then compared the levels of the inhibitory receptors between the subgroups. The results showed that *PDCD1* (mean difference 0.339) (Figure 4A), *KLRG1* (mean difference 0.293) (Figure 4B), *CTLA4* (mean difference 0.843) (Figure 4C), *BTLA* (mean difference 0.958) (Figure 4E), *CD160* (mean difference 0.451) (Figure 4F), and *LAG3* (mean difference 0.78) (Figure 4G) were upregulated in the patients with high level of *HAVCR2/TIGIT* co-expression. As for patients with higher level of *HAVCR2/2B4* co-expression, *PDCD1* (mean difference 0.298) (Figure 4H), *CTLA4* (mean difference 0714) (Figure 4J), *TIGIT* (mean difference 1.353) (Figure 4K), *BTLA* (mean difference 0.777) (Figure 4L), *CD160* (mean difference 0.629) (Figure 4M), and *LAG3* (mean difference 0.874) (Figure 4N) were significantly upregulated (all *P* < 0.05). In contrast, *KLRG1* (mean difference 0.169) was not found to be different (*P* > 0.05) between patients with different *TIM3/2B4* levels (Figure 4I).

## Discussion

T cell-mediated cellular immunity is critical for the antitumor immune response. The CD8+ T cells are antigen-specific killer cells targeting tumor cells in the antitumor immune response. However, CD8+ T cells are often found in an unresponsive state and lose their ability to kill tumor cells in the tumor-induced immunosuppressive microenvironment. The main reason for the suppression is the expression of a variety of inhibitory receptors on CD8+ T cells. The inhibitory receptors affect T cell functions through different signaling pathways, sometimes making it difficult to completely re-invigorate T cell function by targeting individual inhibitory receptors. Naturally, it is worth to explore the synergistic effects by targeting multiple suppressive molecules on the surface of the exhausted T cells. Kuzevanova et al. [20] demonstrated that there is an increased level of TIGIT ligands on the surface of tumor cells when patients are treated with antibodies against PD-1, which may explain why anti-PD-1 alone cannot fully re-invigorate T cell and suppress disease progression in some patients. It also indicates the greater potential of blocking multiple inhibitory receptors at the same time to achieve better antitumor effects.

Studies have shown that the immune escape plays an important role in the pathogenesis of NPC. It would be informative to study the expression pattern of multiple inhibitory receptors so as to design precise treatment against NPC [2]. It is known that a high number of T cells infiltrate the tumor tissue of NPC, which makes it reasonable to develop immunotherapy by exploiting T cells [21]. Currently, disease progression is frequently reported in patients treated with ICIs, which is likely due to resistance to anti-PD-1/PD-L1 and anti-CTLA-4 agents over time [22]. Targeting new inhibitory receptors (TIM-3, TIGIT, 2B4, etc.) [23, 24] is constantly being studied, with combination therapy with immune targets showing positive response efficacy [16, 25]. TIM-3, TIGIT, and 2B4 are co-expressed on T cells in tumor tissue and peripheral blood from multiple types of cancer [26]. However, it remains unclear whether this co-expression also exists in NPC patients. Since TIM-3 is considered a relevant target for combination ICI therapy [27], we decided to investigate the co-expression of TIM-3 with TIGIT or 2B4.

The expression pattern of inhibitory receptors could contribute to the treatment outcome of ICIs. The success of immunotherapy is directly related to the inhibitory receptors level in tumor. Elevated levels of inhibitory receptors co-expression may have a synergistic effect in suppressing T cell function, suggesting that combined ICIs can potentially improve the treatment outcome of immunotherapy [28]. In this study, we found that both TIM-3/TIGIT and TIM-3/2B4 co-expression was higher on peripheral blood CD8+ T cells of patients with NPC than in the healthy population. Our results on TIM-3/TIGIT were consistent with that reported by Runlin et al. [29] in hepatocellular carcinoma. It is suggested that the expression of TIM-3/TIGIT and TIM-3/2B4 may be related to tumorigenesis. In addition, it should be noted that the interaction between 2B4 and CD48 produces both inhibitory and activating signals [15]. However, 2B4 exhibits inhibitory function when highly expressed on CD8+ T cells [30]. In multiple cancer models, the co-expression of 2B4 with other inhibitory receptors indicates the exhaustion of CD8+ T cells [30].

Some studies have reported that the expression levels of immune checkpoint receptors, such as TIM-3, TIGIT, and CTLA-4, increase with human aging [31]. Furthermore, a study by Lee et al. found that the frequency of TIM-3+ PD-1+ CD8+ T cells correlated with age, showing reduced production of inflammatory cytokines such as tumor necrosis factor alpha in older subjects [32]. Our study further confirmed that the co-expressions of TIM-3/TIGIT and TIM-3/2B4 on CD8+ T cells correlated with age (both *P* < 0.05) in that a greater proportion of patients aged ≥ 52 years had higher level of TIM-3/TIGIT or TIM-3/2B4 co-expression. Subsequently, we analyzed the prognosis of patients grouped by age median and found that TIM-3/TIGIT and TIM-3/2B4 co-expression was associated with PFS in NPC patients aged ≥ 52 years (both *P* < 0.05), which was not seen in patients aged < 52 years. The above results suggest that co-expression of TIM-3/TIGIT and TIM-3/2B4 cells in patients with NPC is associated with aging-mediated immune dysfunction and that TIM-3/TIGIT and TIM-3/2B4 promote tumor progression in patients with NPC aged ≥ 52 years.

Sex is reported to be related to different responses to immunotherapy [33]. A meta-analysis of a phase III randomized clinical trials showed that checkpoint inhibitors had more beneficial effect on overall survival and progression-free survival in men. Among these, anti-CTLA-4 was more beneficial in men relative to anti-PD-1/PD-L1 therapy [34]. In this study, we found that TIM-3/2B4 co-expression on CD8+ T cells was correlated with sex, with a greater proportion of males than females being TIM-3/2B4 double positive (*P* ═ 0.014). Similar sex-related difference in co-inhibitory receptor expression has been reported in other advanced malignancies and correlated with prognosis [35, 36]. Blocking immune checkpoint receptors with increased expression could improve therapeutic response to ICIs [21]. Therefore, it is worth further exploring whether treatment benefit is related to sex in immunotherapy for locally advanced NPC targeting co-expression inhibitory receptor targets.

It has been suggested that the number and proportion of tumor tissue-infiltrating T lymphocytes in non-keratinized NPC vary according to the degree of differentiation. Patients with undifferentiated NPC have stronger anti-tumor response elicited by immune cells [37]. Our study further confirmed that the co-expression of TIM-3/TIGIT on peripheral CD8+ T correlated with pathological staging. A higher proportion of patients with undifferentiated (25.3%) than differentiated types (21.7%) (*P* ═ 0.045) had CD8+ T cells co-expressing TIM-3 and TIGIT, suggesting a correlation between increased TIM-3/TIGIT co-expression and immunosuppressive environment in NPC.

TIM-3, TIGIT, and 2B4 were shown to be inversely correlated with disease progression in a variety of tumors [15, 38, 39]. A study on hepatocellular carcinoma found that TIM-3+ TIGIT+ CD8+ T cells were associated with the rate of progression and poor prognosis of patients with hepatocellular carcinoma [29]. Another study reported that co-expression of PD-1 and TIM-3 in colorectal adenocarcinoma may be associated with poor prognosis [40]. Several studies have also found a similar correlation between the co-expression of multiple co-inhibitory molecules and tumor prognosis [17, 41]. However, the prognostic value of TIM-3/TIGIT and TIM-3/2B4 co-expression in NPC remains unclear. Our study confirmed worse OS in the patients with higher co-expression of TIM-3/TIGIT or TIM-3/2B4 on CD8+ T cells. TIM-3/TIGIT co-expression was associated with PFS and DDFS in patients with NPC. Moreover, TIM-3/TIGIT co-expression was an independent prognostic factor for OS in NPC, whereas smoking was an independent prognostic factor for PFS. It was suggested that TIM-3/TIGIT co-expression level was associated with poor prognosis of NPC more than that of TIM-3/2B4. The prognostic effects of high expression of TIM-3/TIGIT and TIM-3/2B4 suggest that these two can be prognostically effective predictors and can be developed as novel combination therapy targets.

Exhausted CD8+ T cells have been reported to co-express multiple inhibitory receptors, the pattern and number of which correlate with the level of T cell exhaustion [42]. The pattern of co-expression and the number of inhibitory receptors co-expressed by the same CD8+ T cells can significantly affect the severity of dysfunction [43]. In this study, CTLA-4 was also found to be highly expressed on the CD8+ T cells with higher level of TIM-3/TIGIT or TIM-3/2B4 co-expression (*P* < 0.05). Analysis using mRNA data from the GEO database also suggested that the expression of *PDCD1, CTLA4, CD244, BTLA, CD160*, and *LAG3* was upregulated when mRNA of *TIM3/TIGIT* or *TIM3/2B4* was highly expressed, which is consistent with the results of a study on Hepatocellular carcinoma [44], where cells with elevated PD-1/TIGIT co-expression also had increased expression of TIM-3, LAG-3, and 2B4. Our study confirmed that the expression levels of inhibitory receptors were also increased when TIM-3/TIGIT or TIM-3/2B4 were highly expressed. Some of the differences in the results between the flow data and GEO database-derived data may lie in the inconsistency between protein expression and mRNA. In summary, by analyzing the correlation between T cell inhibitory receptor co-expression and the expression profiles of several other inhibitory receptors, we comprehensively evaluated the pattern of immune checkpoint receptors on peripheral CD8+ T cells in patients with NPC to more effectively guide the selection of inhibitors against immune checkpoint receptors for combination therapies.

There are some limitations to this study. Our results are based on an examination of PBMCs rather than on tumor tissue. It is known that the tumor microenvironment greatly affects the phenotypes of T cells. It is worthwhile to validate our results by studying tumor-infiltrating lymphocytes regarding the co-expression pattern of the immune checkpoint receptors. In addition, some patients receiving salvage treatment require further research among a larger cohort.

## Conclusion

In this study, three inhibitory receptors, TIM-3, TIGIT, and 2B4, on CD8+T cells were investigated as new immunotherapeutic targets for NPC. The co-expression of TIM-3 with TIGIT or 2B4 was studied to discover the correlation between inhibitory receptor co-expression and clinical characteristics and prognosis, providing a theoretical basis for the development of potential immune targets for combined immunotherapy.
